# Stakeholder’s experience on financial incentive-based regulatory instruments for public-private partnership in developing countries: a study from Iran’s primary health care delivery system

**DOI:** 10.1017/S1463423626101431

**Published:** 2026-07-10

**Authors:** Iman Keliddar, Amin Torabipour, SeyedMehdi EmamianFard

**Affiliations:** Ahvaz Jundishapur University of Medical Sciences: Ahvaz Jondishapour University, Islamic Republic of Iran

**Keywords:** financial incentives, Iran, PHC, public-private partnership, regulatory instruments

## Abstract

**Background:**

Regulatory instruments are a key necessity to implement public-private partnership’s strategy. This study aimed to explore the stakeholders’ experience on financial incentive-based regulatory instruments for public-private partnership in Iran’s primary health care (PHC) delivery system.

**Methods:**

This qualitative study was involved face-to-face interviews with 18 stakeholders in primary health care partnership projects including employers, experts, contractors, and executive managers of contracted companies operating as a private health sector participant in primary health care services. The data were analyzed using the framework analysis method.

**Results:**

Twenty-four codes were developed. Findings showed that the current state of financial incentive-based regulatory instruments in Iran’s PHC delivery system faced some challenges despite existing capacities. These challenges include the lack of an independent trustee for access to capital, and a comprehensive regulatory program to facilitate private sector participants’ access to capital, and partnership contracting mechanisms. Findings also showed main challenges of these instruments related to access to capital, tax incentives and subsidies, staff mobility control mechanisms, partnership contracting mechanisms, and provider payments.

**Conclusion:**

The presence of significant challenges in Iran’s health care system can impact the private sector’s motivation to participate in primary health care. By improvement the infrastructure, reforming legal processes, and providing financial incentives, the government can boost the private sector’s motivation in primary health care and advance the health sector’s goals.

## Background

Health care services in Iran are provided at three levels. The first level includes health houses and comprehensive health centers (CHC), which provide primary health care in rural and urban areas. The second level consists of units that are able to provide more specialized medical services, such as regional health center, regional hospitals, and specialized outpatient clinics. The third level includes specialized clinical and educational services that complement the second level including specialized and sub-specialized hospitals, the provincial health center, and various medical schools. Patients at these levels receive services through a referral system (Gharaee *et al*., 2023).

Global experiences demonstrate that enhancing primary health care is the most efficient strategy to accomplish the objectives of the health system. Participation stands as one of the key pillars of primary health care. To cultivate this partnership, the public-private partnership strategy should be implemented. The potential of the private sector in health promotion remains underutilized in numerous countries (Baig *et al*., 2014). In fact, Public-Private Partnership (PPP) is a bilateral partnership and win-win policy between the public and private sectors. In this partnership, the interests of both parties should be considered. The public sector provides infrastructure and monitoring services, while the private sector contributes new capacities and resources (Tabrizi *et al*., 2020). Provision of PHC services solely via the public sector providers has its limitation and potential problems in response to challenges of health services delivery; public-private partnerships (PPPs) initiatives could help to make PHC services provision more effective and efficient. PPPs are voluntary cooperative arrangements between two and more public and private sectors in which all participants agree to work together to achieve a common purpose or undertake a specific task and to share risks and responsibilities, resources, and benefits (Ardian *et al*., 2007). For example, among the objectives of PPPs could be the establishment of a sustainable financial system, capacity-building, cost control, and improving the health of the community (Joudyian *et al*., 2021). Limited resources, high costs, and low-quality services provided in the public sector cause many governments worldwide to seek the use of the capital and expertise capacity of the private sector to minimize their infrastructure deficiencies (Nikniaz *et al*., 2006; Osei-Kyei and Chan, 2015). One way of utilizing the private sector’s capacities is through aligned participation with public bodies to achieve common goals, conceptualized as ‘public-private partnership’ (Zarei *et al*., 2018). Creating favorable conditions for implementing public-private partnerships and using all effective instruments to develop its required infrastructure is one of the crucial tasks of governments. This issue has also been emphasized in world forums. At the ESCAP Conference, it was emphasized that countries should utilize all influential factors and instruments to develop infrastructure for public-private participation and work to create or improve favorable conditions for such partnerships (Llanto *et al*., 2015).

Given the private sector’s potential interest in increasing profit and efficiency, it is necessary for governments to utilize regulatory instruments to harness the private sector’s capacity to achieve social goals (Smith *et al*., 2001; Harding and Preker, 2003; Halabi, 2019; Motamedi *et al*., 2021). One key infrastructure for providing objectivity to public-private partnerships is the use of regulatory instruments (Alijani and Joneydi, 2020). Health system governors should gather and assess information about their country’s regulatory instruments and mechanisms for public-private partnerships in order to address any issues and move forward with their partnership initiatives (Harding and Preker, 2003).

A set of regulatory instruments is utilized to establish an incentive environment for participatory activities aimed at achieving public health goals among healthcare providers. Various regulatory incentives have been implemented worldwide. Since the 1990s, the use of these instruments, known for their soft and voluntary nature, has been widely embraced. One category of these incentive instruments seeks to influence the behavior of healthcare service providers towards specific targets by implementing financial mechanisms (Harding and Preker, 2003).

However, as concluded from national and international records, despite the increasing use of partnership incentive-based instruments to improve the performance of the health system, aligning these instruments with the goals of the health system and redesigning them to support participatory approaches seems vital (Custers *et al*., 2008). Studies have shown that continuous monitoring and sophisticated designations are necessary to use these incentive-based instruments to influence the behavior of health services providers in line with public health goals (Roy, 2016; Zegraoui *et al*., 2018). Therefore, regulatory reform is essential to ensure that participatory initiatives align with the health goals of the population.

The experience of participatory activities in the primary health care delivery system in Iran began in 2014 with the implementation of the Health Transformation Plan. Since then, no study was conducted examining the status of financial incentive-based regulatory instruments for public-private partnership in primary healthcare in Iran as a developing country. In addition, much empirical literature on public-private partnerships was devoted to high-income countries, and there was very little information on the performance of these partnerships in developing countries (Hellowell, 2019). Therefore, this study explores stakeholders’ experiences with financial incentives regulating public-private participation in Iran’s primary healthcare delivery system. The study investigates the capacity of regulatory structures in Iran’s health sector in terms of regulatory instruments for financial incentives in the provision of primary healthcare services, using the Kumarayanke framework (Kumaranayake, 1997).

The results of this study could provide a conceptual framework to strengthen the primary health care (PHC) sector in Iran, a developing country, in achieving community health goals. Additionally, it could offer valuable information to national and local governments regarding the design of financial regulatory instruments for Iran’s primary health care delivery system.

## Methods

### Study participants

In this qualitative study, the samples consisted of stakeholders actively involved in primary health care partnership projects in Khuzestan, Iran. This included Deputies of Health (DH) in Universities of Medical Sciences (UMSs, and the units’ Executive managers), experts (legal and contract experts, and professors with expertise in outsourcing), as well as contractors and executive managers of contracted companies working in the private health sector participating in primary health care services. The purposeful and snowball sampling methods were used to select the study participants. In our study, employers with at least three years of managerial experience or decision-making authority in public-private partnerships activities, professionals with at least one study on public-private partnership issues or knowledge about the subject and teaching in the field, and contractors with a license or experience in private sector companies participating in the provision of primary health care participated.

In all of the above cases, the exclusion criteria were the individuals’ inability or unwillingness to participate in the study. The sampling process continued until data saturation was reached. The interviews began after necessary permissions were obtained and key participants in the Khuzestan DH were identified. Additional interviewees were included in the study using the snowball method if they met the inclusion criteria.

### Data collection

In this study, Kumarayanke’s framework, later provided by the World Bank, was chosen as the study framework for analyzing financial regulatory instruments for public-private partnerships due to its relevance to health services (Kumaranayake, 1997; Smith *et al.*, 2001). The framework was used to create an interview guide. Three in-depth semi-structured interviews were then conducted to assess the interview guide and address any ambiguous items. The interview participants included an employer, an expert, and a partnership contractor.

After compiling the interview guide, face-to-face interviews were conducted. At the start of each interview, the researchers explained the study objectives to the participants, who were interviewed after providing informed consent. All interviews took place at the interviewees’ workplaces. Interviews continued until data saturation was reached.

### Data analysis

All interviews were recorded and transcribed immediately. The framework analysis method, mainly used in policy studies, was applied to analyze the data. This method consists of five stages: familiarization, identifying a thematic framework, indexing, charting, and mapping, and interpretation (Nikpeyma *et al*., 2014). During the familiarization stage, the researchers immersed themselves in the data by listening to audiotapes, taking notes during the interviews, transcribing the interviews, and providing a summary of them. The research team gained an overview of the data, identified critical ideas and frequent themes, and wrote them down in the margins. They then checked the collected data against the research objectives to ensure alignment. In the second stage, the World Bank model was identified as the thematic framework to organize the key ideas and repeated themes from the introduction phase. Repetitive ideas were grouped as similar and thematic ideas. In the indexing stage, each interview was individually coded to examine the structure, implementation instructions, and challenges of the current state of the primary healthcare delivery system in terms of the five areas of financial regulatory instruments for public-private partnerships. The research team extracted a list of text codes based on the thematic framework. After indexing the data, the team summarized it in thematic tables to easily visualize all the data. In the final stage, the team reviewed all the tables, compared participants’ viewpoints and interpretive patterns, and prepared an explanatory account of the data. The thematic framework was revised multiple times throughout the analysis process.

### Quality considerations

The distinction between the stages of the framework analysis method facilitated the transparency of the data analysis process and strengthened the data rigor. Each of the stages and the decisions taken through could be easily observed and corrected if necessary. At any stage of the analysis process, the researchers could trace back to the original data and thus facilitate the inductive and iterative approach that is a feature of a qualitative study (Furber, 2010).

## Results

The total number of participants was 18, with 3 being contractors, 4 being specialists, and 11 being employers. A total of 24 codes were developed. The findings from the stakeholders’ views and experiences indicated that the current regulatory instruments related to financial incentives have both strengths and challenges. These include facilitating access to capital, tax incentives and other subsidies, staff mobility control mechanisms, partnership contracting mechanisms, and provider payments. This situation can influence the motivation of private sector partnerships in providing primary health care services (see Table 1).

**Table 1.** Financial incentive-based regulatory instruments for public-private partnership in Iran’s PHC delivery system Table 1 long description.

Themes	Sub-themes	Codes
Access to capital	Stewardship	Lack of an independent trustee
	Program	Lack of a comprehensive regulatory program to facilitate private sector participants’ access to capital
	Challenges	Lack of low-cost or long-term loans
		Lack of government guarantees
		Lack of access to foreign currency
		Complexity of administrative procedures
Tax incentives and other subsidies	Stewardship	Deputy of Health in the MOHME
	Program	Tax exemption guidelines for health service cooperatives
	Challenges	Administrative procedures Inconsistency
		Policy considerations in private sector tax exemptions
Staff mobility control mechanisms	Stewardship	Deputy of Health in the MOHME
	Program	Instructions for financial incentives for activities in disadvantaged urban and rural areas
	Challenges	Lack of adequate financial support
Partnership contracting mechanisms	Stewardship	Deputy of Health in the UMSs
	Program	Lack of a single instruction
	Challenges	The haste in concluding partnership contracts
		Weakness in the regulated partnership contracts
		Weakness in the expert capacity required for drafting partnership contracts
		Viewpoints inconsistency on the setting of partnership contracts
Provider payment	Stewardship	Deputy of Health in the UMSs
	Program	Existence of instructions
	Challenges	Delays in fulfilling government obligations
		Low-profit margins
		Sustainability of financial resources

### Access to capital

According to the participants, there was no independent trustee available for private sector participants to access capital markets. Additionally, the lack of a comprehensive program to facilitate access to capital was identified as a major shortcoming in this area.

‘Contractors in the Health Transformation Plan do not receive any financial support’ (P2).

The findings also revealed that private sector participants faced challenges in accessing capital markets due to the absence of low-cost or long-term loans, government guarantees, access to foreign currency, and the complexity of administrative procedures.

#### Lack of low-cost or long-term loans

According to the participants, private companies collaborating with the health sector have not had access to long-term and low-cost loans.

One participant stated, ‘Until now, they haven’t offered us low-cost and long-term loans’.

These companies could potentially utilize the regular loans offered by financial institutions based on their financial performance in banks and credit institutions. However, the availability of these loans was not contingent on the nature of collaboration for active private companies, and there was no specific advantage for those involved in primary healthcare.

#### Lack of government guarantees

The findings revealed that government guarantees were not provided to facilitate access to capital markets for private sector participants. Additionally, the public sector received bank guarantees and an amount known as ‘Hosne Anjame Kar’ for good quality performance in exchange for their involvement in providing primary health care.

‘We deposited a large sum of money in the bank as a guarantee for our contract, and our money remained untouched for nearly a year’ (P2).

‘According to the Financial and Transaction Regulation, the company must deposit 10% of the contract fund amount in the bank as a bank guarantee, known as ‘Hosne Anjame Kar’, and provide a promissory note to the university. This amount is only released at the end of the contract if no issues arise between the employer and the company’ (K3).

The heavy bank guarantees and upfront deductions led to a decrease in the private sector’s capital due to inflation. Moreover, the bank procedures for obtaining such guarantees were extremely cumbersome.

‘At times, it takes companies two months to secure a bank guarantee because banks impose difficult conditions, and some banks refuse altogether’ (M4).

#### Lack of access to foreign currency

According to the findings, foreign currency was not given to private sector participants. However, given that private sector participation in primary health care was often in the staffing field, there was no need to provide foreign currency to private sector participants.

‘In general, we’re human resources contractors, and the employer provides us with technical equipment himself. So, there’s no need for foreign currency indeed. We only handle the public sector staffing’ (P2).

#### The administrative procedures complexity

The participants argued that administrative procedures for private sector’s access to capital markets, tax exemptions, and other assistance were fraught with ambiguities and inconsistencies. Sometimes there were inconsistencies between different stakeholders regarding the procedures for granting private sector participation tax exemptions.

‘When companies wanted to take recoupment accounts, the tax office deducted 9% VAT from them all. They said they had been told they would be exempted from 9% VAT, but the tax office took the money from them’ (M3).

Sometimes the new rules were inconsistent with the current capacities of private sector participants in the business environment, which made some challenges for law enforcement procedures.

‘A service company article of association might have been approved 10 to 15 years ago. Well, there was no health sector privatization at all in the last 15 years’ (M4).

### Tax incentives and other subsidies

According to the participants, the Ministry of Health was in charge of tax incentives and other subsidies. There were also tax exemption guidelines for companies called ‘Health Services Cooperatives’.

‘Health services cooperatives that do only health work are tax-exempt’ (K3).

However, there were no guidelines for insurance payments and other exemptions.

‘We have to pay urban tolls for our rental places and insurance. If we don’t pay on time, we’ll be fined’ (P1).

Under other general laws, companies supplying manpower and equipment were tax-exempt. However, these exemptions did not depend on the companies’ participation models. Besides, working in primary healthcare did not consider a unique advantage in this regard.

‘Companies that supply manpower are exempt from VAT’ (M3).

#### Administrative procedures inconsistency

As the participants discussed, despite receiving instructions on tax exemptions, they were facing challenges in implementation due to inconsistencies in administrative procedures. This inconsistency in administrative procedures was causing difficulties in applying tax exemptions, highlighting the need for more attention to policy considerations.

‘It’s the different interpretations of these laws that cause issues. Even the enforcers of these laws have their own unique interpretations, leading to situations where exemptions are sometimes not granted’ (M3).

#### Policy considerations in private sector tax exemptions

On the one hand, the participants noted the importance of tax revenues as one of the government’s most important sources of revenue. On the other hand, they believed that long-term tax breaks could be an essential incentive to attract some in areas that provided public services such as primary healthcare.

‘I think paying taxes on current government spending is necessary, but well, if some waivers or exemptions are provided for health companies, it will cause investment direction in this area’ (k3).

The participants also argued that in order for these tax waivers or exemptions to expand private sector participants’ investments in the health sector for the long run, their implementation should comply with policy considerations.

‘Given that the purpose of paying taxes is to provide public services to the populations, I think taxes should be levied. But I also believe that in general, services that benefit the public should pay fewer taxes, and instead, more taxes should be paid on harmful goods and services’(M4).

### Staff mobility control mechanisms

The findings showed some financial incentives set for the private sector participants providing primary healthcare services for disadvantaged areas in urban and rural populations in some current instructions.

‘There is a deprivation coefficient for rural areas, only in the rural insurance sector. The higher the deprivation coefficient, the more you pay the doctors and the lower the tax’ (k3).

‘According to the urban guideline, the price of services in informal settlements is slightly higher to motivate health care providers’ (M2).

#### Lack of adequate financial support

According to the findings, although there were national instructions for regulating the application of financial incentives in deprived areas, their implementation was not the same in urban and rural populations. In the urban sector, implementing these instructions was facing challenging due to the lack of adequate financial support. Thus, it could be concluded that controlling staff mobility in the urban sector had not been successful in practice.

‘The price of services in informal settlements intended to motivate healthcare providers a little more, but it didn’t happen due to the lack of financial resources and a regular financial system for payments’ (M2).

### Partnership contracting mechanisms

The participants said that DH in Universities of Medical Sciences (UMSs) were responsible for arranging and concluding the partnership contracts in providing primary healthcare to geographical areas they covered.

‘The authority to arrange the contract is delegated in the provinces themselves’ (p3).

The participants’ statements also showed no unique instruction for concluding contracts that could be used as a basis for arranging partnership contracts’ contents in primary healthcare. They believed that the most critical challenges of the contracting mechanisms were the haste in concluding contracts, the weakness of regulated contracts, the lack of expert capacity required for drafting partnership contracts and their contents for the provision of primary healthcare services, and the inconsistency of the viewpoints on partnership contracts in primary healthcare services.

#### The haste in concluding partnership contracts

One of the challenges of concluding partnership agreements in delivering primary healthcare was the haste in concluding the PHC partnership contracts.

‘We want to do something in a hurry, but then we stay inside with numerous problems’ (M4).

#### Weakness in the regulated partnership contracts

The current partnership contracts in the provision of primary healthcare were weak and did not meet the required standards.

‘There are always some contradictions in PHC contracts. Many of the issues the employers ask us about aren’t mentioned in the contract’ (P1).

#### Weakness in the expert capacity required for drafting partnership contracts

According to the findings, the current capacity of the experts drafting and concluding partnership contracts in the provision of primary healthcare was weak. The current expert capacity was not sufficient to set professional drafts of partnership contracts.

‘The contract issue is a new system added to our job, and we don’t yet have professionals in this field’ (k1).

#### Viewpoints inconsistency on the setting of partnership contracts

The interviewees’ statements indicated the lack of a unified view on the arrangement of partnership contracts in the provision of primary healthcare. One example of this is the lack of coordination between various deputy stakeholders, and even between the local (Universities of Medical Sciences) and national (Ministry of Health) areas regarding contract qualifications. This creates severe challenges in this regard.

‘There’s no single contract framework in the country. We’ve got an expert who prepares a contract according to the national instructions, and the Contract Affairs Unit approves it’ (k1).

### Provider payments

According to the findings, DH in UMSs were responsible for private sector participants’ payments in primary healthcare. There were also some national instructions about the private sector partnership payment arrangements.

‘Our payment system guidelines are the same as the urban plan instruction issued by the Ministry of Health’ (M1).

Based on the ‘National Instruction for Providing Primary Health Care in Urban Areas’, the private sector participants’ payment mechanisms and methods were different according to the partnership contract model. In this regard, there were two models of concluding partnership contracts, i.e., Outsourcing and Services Purchasing. In the former, the partnership contract payment was based on the Capitation method, and in the latter, the payment system would be based on the Pay for Performance method.

‘The payment system depends on whether you have fully outsourced or purchased the services’ (M1).

The payment system of primary healthcare partnership was facing some challenges in terms of delays in fulfilling government obligations, low-profit margins, and sustainability of financial resources.

#### Delays in fulfilling government obligations

The research findings showed that the public sector was late to fulfill its financial obligations to the providers.

‘I think the public sector doesn’t act well in most of its obligations to contractors, and payments are delayed’ (M3).

#### Low-profit margins

According to the participants, one of the main challenges in providers’ payments was the weakness in considering unexpected events, which, when concluding partnership contracts, ensured a reasonable profit margin.

‘Currently, due to the lack of a good profit margin for contractors, unfortunately, most contractors run away after the end of the contract and invest their capital elsewhere’ (M1).

#### Sustainability of financial resources

The participants believed that the sustainability of financial sources of payments for providers was weak and depended on conditions. This had a negative effect on payment for services provided by the private sector.

‘The ministry reduces the credits due to the economic conditions, and doesn’t send the credits on time’ (K7).

## Discussion

This study explored the lived experiences of stakeholders regarding financial incentives and regulatory instruments aimed at increasing private sector participation in the provision of primary health care services in Iran. Reforms to strengthen Iran’s primary health care delivery system, such as facilitating access to capital, providing tax incentives and subsidies, implementing staff mobility control mechanisms, establishing partnership contracting mechanisms for primary health care delivery, and improving provider payments, could enhance the effectiveness of these crucial financial incentive regulatory instruments. By enhancing the current structures, procedures, and addressing challenges in these instruments, we can boost private sector motivation for participating in the provision of primary health care services.

### Access to capital

One of the financial incentives to attract private sector participation in partnership projects is to facilitate their access to the capital they need. Many investments in the health sector require access to significant capital as financing sources to increase capacities in buildings, equipment, and other start-up costs (Harding and Preker, 2003; Hashim *et al*., 2016). The findings showed that there was no independent trustee to facilitate private sector participants’ access to capital markets. In line with these findings, the study by Rasi *et al.* also indicated that one of the challenges of privatization in Mashhad was the lack of an independent organization responsible for decision-making in outsourcing (Rasi *et al*., 2018). The study by Ozorhon *et al.* identified the most critical challenge for private sector participation in healthcare delivery in developing countries as the lack of legal frameworks and institutional capacity, banking payment mechanisms, and appropriate regulatory systems (Ozorhon *et al*., 2021).

The absence of an independent trustee results in gaps in the comprehensive regulatory process that facilitates private sector participants’ access to capital markets. This issue, highlighted in various national and provincial studies, is a significant shortcoming in this area. In a study by Hamidizadeh *et al.* the lack of a clear and stable legal framework was identified as one of the critical factors contributing to the failure of public-private partnerships in the Iranian pharmaceutical industry (Hamidizadeh *et al*., 2019). In a study by Karimi *et al.* the lack of necessary infrastructures in the country’s market, culture, and organizations was found to be a barrier to outsourcing implementation in the health sector (Karimi *et al*., 2011). Danaeifard *et al.* identified obstacles to developing public-private participation in national projects, including the lack of clear legal frameworks and transparency in the public sector, which led to financial problems and a lack of liquidity in the private sector (Danaie Fard *et al*., 2017). Similarly, Mohammadi *et al.* reported factors such as the absence of a proper mechanism to utilize banking facilities from the National Development Fund and the Islamic Development Bank, which were the most critical obstacles to financing public-private partnership contracts (Mohammadi *et al*., 2020).

Government support and ongoing oversight of flexible financial instruments, such as lending, low tax rates, and granting credit for suppliers, have been cited as crucial factors in the success of public-private partnerships in other studies (Hamidizadeh *et al.*, 2019). This study highlights some challenges in facilitating access for the private primary healthcare delivery sector to capital markets. These challenges are mainly related to the lack of low-cost and long-term loans, government guarantees, access to foreign currencies, and complex administrative procedures. The study by Rathi *et al.* also reported the existence of complex and lengthy bureaucracies in the outsourcing processes of Mashhad city (Rasi *et al.*, 2018). Some challenges identified in this study have been reported in other countries as well. A study in Malaysia identified the lack of support for public-private partnership programs and the difficulty of accessing long-term financial resources as critical obstacles to the success of partnership projects (Ismail, 2013). Gomez (2004) identified macroeconomic fluctuations in currency and purchasing power as severe obstacles to privatization (Gómez-Ibáñez *et al*., 2004). Another study conducted in Saudi Arabia in 2020 identified problems related to administrative procedures and instructions, changes in laws and regulations, weaknesses in regulatory and enforcement frameworks, issues in receiving credits from banks, and local institutions’ inability to provide long-term financing as critical obstacles to the development of partnership projects in the health sector (Al-Hanawi *et al*., 2020). The lack of access for the private sector to capital markets has severe consequences for developing investments in the provision of primary healthcare. Additionally, receiving heavy bank guarantees and simultaneously deducting money in advance as a performance incentive puts double pressure on the private sector and may decrease the private sector’s capital due to inflation. Therefore, there is a crucial need for reforms such as establishing independent units to address issues related to regulating primary healthcare partnership projects, developing comprehensive legal programs to facilitate access to long-term and low-cost loans for private sector participants in primary healthcare (Health Services Cooperatives), removing barriers for granting government guarantees, and reducing the complexity of administrative procedures. These reforms can facilitate the private sector’s access to the funds needed for necessary funding and increase willingness to participate in primary healthcare delivery projects.

### Tax incentives and other subsidies

Tax exemptions are crucial incentives that influence the behaviors of the private sector in support of public health policies and are highly attractive to the professional and business community (Custers *et al.*, 2008). Some studies have suggested that the most significant obstacle to the success of public-private partnership efforts is the government’s failure to fill financial gaps through incentives and subsidies (Ismail, 2013). Participants’ statements indicated that centralized instructions were developed to enforce tax incentives and other subsidies for a group of companies known as ‘Health Services Cooperatives.’ However, these instructions did not include insurance payments and other exemptions Gayum zadeh *et al.* identified cumbersome insurance and tax laws as barriers to service delivery in Tehran’s participatory health centers (Gayum Zadeh *et al*., 2018).

However, the implementation of tax incentives and subsidies is sometimes challenged by existing administrative inconsistencies or inconsistencies with the current capacities of private sector participants. Mohammadi *et al*. addressed the lack of sufficient capacity to define the incentives and exemptions in partnership schemes (Mohammadi *et al.*, 2020). The lack of government support for tax settlements was also reported in a 2007 study by Chen *et al.* (Chan *et al*., 2006). Therefore, reforms in areas such as the compatibility of tax regulation activities with existing capacities and laws are suggested to facilitate the required coordination between different institutions to implement tax policies. Another challenge with tax exemptions is paying more attention to policy considerations. Since tax revenues have significant potential uses, the value of the benefits of these financial regulatory incentives should be weighed against the cost of projected revenues if they are received. Any incorrect design and implementation of policies in this area can lead to corruption and disrupt the national tax balance. Therefore, for tax exemptions to create attractiveness for the development of private sector participants’ investments in areas such as primary healthcare in the long run, their enforcement should take into account policy-making considerations and the benefits of enforcing them in primary healthcare.

### Staff mobility control mechanisms

Governments use a wide range of mechanisms to control staff mobility. The use of staff mobility control mechanisms can help attract the cooperation of private sector participants in partnership projects. The findings showed that despite financial incentives for activities in disadvantaged areas in national instructions, the quality of their implementation in urban and rural populations was unequal. Accordingly, the implementation of regulatory activities to control staff mobility, especially in urban populations, has not been successful in practice. This has been mainly due to the lack of adequate financial support for implementing regulations, which has made it difficult for the urban sector to implement and enforce these incentives. Despite numerous initiatives taken by the government to establish health equity and the population’s integrated and fair access to health services, including the expansion of health services in deprived areas, unfortunately, the health equity problems are still apparent. Also, the lack of appropriate incentives to work in disadvantaged regions has made the private sector reluctant to provide care services to these regions (Mir *et al*., 2019). Private sector participation can be beneficial in addressing this challenge and improving the health equity situation. Therefore, it is necessary to consider sustainable financial resources and enforce the regulations in staff mobility control mechanisms, especially in urban areas, to better exploit the benefits of private sector participation in deprived regions. Therefore, considering sustainable financing mechanisms, measures like participation of third-party payers such as insurance companies is recommended in urban areas.

### Partnership contracting mechanisms for the provision of primary health care

The regulation of partnership contracting mechanisms is a powerful incentive tool that impacts the provision of primary healthcare services by healthcare providers (Hashim *et al.*, 2016; Ozorhon *et al.*, 2021). According to the findings, DH in UMSs are responsible for organizing and finalizing partnership contracts to deliver primary healthcare services in their designated geographical areas. However, there is currently no standardized guideline for the essential components of primary healthcare partnership contracts.

The most critical challenges of primary healthcare contracting mechanisms found in this study were the haste in concluding contracts, the weakness of regulated contracts, the lack of expert capacity required for setting partnership contracts’ contents, and the inconsistency of viewpoints on partnership contracts. According to the findings, the current partnership contracts’ contents could not meet the required standards. In addition, the current expert capacity was not sufficient to develop professional contract contents in the field. Similarly, Danaeifard *et al.* identified the lack of clear contracts, the lack of experts and professionals, and the lack of public sector oversight on technical issues about public-private partnership projects as obstacles to the development of partnership projects and success in public-private partnership strategy (Danaie Fard *et al.*, 2017). Torabipour *et al.* mentioned the main barriers to outsourcing implementation as follows: the lack of outsourcing laws’ transparency, the shortcomings in drafting contracts and transparent mechanisms for public sector oversight on the performance of private sector participants, and the lack of knowledge of managers and experts working in regional trustees of corporate contracts. These had led to problems such as insufficient care for the private sector’s provided services quality and fulfilling the public sector’s goals in drafting participatory contracts (Alizadeh and Torabipour, 2018). The lack of experts familiar with professional competencies in designing and implementing collaborative projects was reported in other studies (Al-Hanawi *et al.*, 2020; Ozorhon *et al.*, 2021). The present study also showed no coordinated alignment among different stakeholder deputies’ views and national and local levels drafting partnership contracts in participation in primary healthcare. This fact has been reported in some other countries as well. For example, in Saudi Arabia, the lack of a single law on contracting and the transparency of partnership processes were identified as the most critical obstacles for public-private partnerships (Al-Hanawi *et al.*, 2020). A study in Iraq in 2019 identified some obstacles such as the lack of a uniform law, private cooperatives shortcomings, inadequate public management processes, and the lack of transparency in administrative processes in the public sector (Rezouki and Hassan, 2019). The weakness in legal frameworks for participatory activities was also reported in the study by Akampurira (Akampurira *et al*., 2009). Overall, this study showed many challenges in the field of partnership contracting mechanisms in the delivery of primary healthcare. It is suggested to train professionals in fields such as partnership contracts’ draft development and implementation of primary healthcare delivery participatory programs. This will create and strengthen the expertise capacity in preparing drafts and concluding partnership contracts. In addition, to standardize the contents and processes of setting up primary healthcare partnership contracts, it is necessary to unify the procedures and perspectives on drafting partnership contracts. Establishing an organizational unit as a trustee to address issues related to regulating primary health care partnership projects is an essential institutional solution in strengthening the mechanisms of partnership agreements. These initiatives can all lead to the effectiveness of regulating and concluding partnership contracts in providing primary healthcare under its specific characteristics.

### Provider payments

The most critical challenges of primary healthcare contracting mechanisms found in this study were the haste in concluding contracts, the weakness of regulated contracts, the lack of expert capacity required for setting partnership contracts’ contents, and the inconsistency of viewpoints on partnership contracts. According to the findings, the current partnership contracts’ contents could not meet the required standards. In addition, the current expert capacity was not sufficient to develop professional contract contents in the field. Similarly, Danaeifard *et al.* identified the lack of clear contracts, the lack of experts and professionals, and the lack of public sector oversight on technical issues about public-private partnership projects as obstacles to the development of partnership projects and success in public-private partnership strategy (Danaie Fard *et al.*, 2017). Torabipour *et al.* mentioned the main barriers to outsourcing implementation as follows: the lack of outsourcing laws’ transparency, the shortcomings in drafting contracts and transparent mechanisms for public sector oversight on the performance of private sector participants, and the lack of knowledge of managers and experts working in regional trustees of corporate contracts. These had led to problems such as insufficient care for the private sector’s provided services quality and fulfilling the public sector’s goals in drafting participatory contracts (Alizadeh and Torabipour, 2018). The lack of experts familiar with professional competencies in designing and implementing collaborative projects was reported in other studies (Al-Hanawi *et al.*, 2020; Ozorhon *et al.*, 2021). The present study also showed no coordinated alignment among different stakeholder deputies’ views and national and local levels drafting partnership contracts in participation in primary healthcare. This fact has been reported in some other countries as well. For example, in Saudi Arabia, the lack of a single law on contracting and the transparency of partnership processes were identified as the most critical obstacles for public-private partnerships (Al-Hanawi *et al.*, 2020). A study in Iraq in 2019 identified some obstacles such as the lack of a uniform law, private cooperatives shortcomings, inadequate public management processes, and the lack of transparency in administrative processes in the public sector (Rezouki and Hassan, 2019). The weakness in legal frameworks for participatory activities was also reported in the study by Akampurira (Akampurira *et al.*, 2009). Overall, this study showed many challenges in the field of partnership contracting mechanisms in the delivery of primary healthcare. It is suggested to train professionals in fields such as partnership contracts’ draft development and implementation of primary healthcare delivery participatory programs. This will create and strengthen the expertise capacity in preparing drafts and concluding partnership contracts. In addition, to standardize the contents and processes of setting up primary healthcare partnership contracts, it is necessary to unify the procedures and perspectives on drafting partnership contracts. Establishing an organizational unit as a trustee to address issues related to regulating primary health care partnership projects is an essential institutional solution in strengthening the mechanisms of partnership agreements. These initiatives can all lead to the effectiveness of regulating and concluding partnership contracts in providing primary healthcare under its specific characteristics.

## Limitations

This is the first study in the field of public-private partnership infrastructure in the field of primary health care in Iran. There were some limitations in our study. First, our findings only reflect outcomes of participation in PHC and did not include health services at other levels. Second, the number of experts and contractors in this field was very small. It was also very difficult to involve these people in this research. Third, the majority of participants were affiliated with private health sector companies. Fourth, another limitation of our study was related to the World Bank model as the thematic framework. Other models can be used to fix the defects of this model.

## Conclusion

The presence of some serious challenges in Iran’s health care system can affect the motivation of the private sector to participate in primary health care. By strengthening the infrastructure, reforming the legal processes and providing financial incentives, the government can increase the motivation of the private sector in primary health care and promote the goals of the health sector. Iran’s financial Incentive-based Regulatory Instruments for Public-Private partnerships in the PHC delivery system, despite existing capacities, faced some challenges. These challenges were related to stewardship, existing a comprehensive program, and implementation challenges. Solving these challenges can be effective in improving the primary health care system. Therefore, there is a fundamental need to modify regulatory tools based on financial incentives to implement public-private partnerships in developing countries such as Iran.

## Data Availability

All data generated or analyzed during this study are included in this published article.

## Table 1. Long description

A table with five themes and their respective sub-themes and codes. The themes are Access to capital, Tax incentives and other subsidies, Staff mobility control mechanisms, Partnership contracting mechanisms, and Provider payment. Each theme has sub-themes under Stewardship, Program, and Challenges. Access to capital includes codes like Lack of an independent trustee, Lack of a comprehensive regulatory program, Lack of low-cost or long-term loans, Lack of government guarantees, Lack of access to foreign currency, and Complexity of administrative procedures. Tax incentives and other subsidies include codes like Deputy of Health in the MOHME, Tax exemption guidelines for health service cooperatives, Administrative procedures Inconsistency, and Policy considerations in private sector tax exemptions. Staff mobility control mechanisms include codes like Deputy of Health in the MOHME, Instructions for financial incentives for activities in disadvantaged urban and rural areas, and Lack of adequate financial support. Partnership contracting mechanisms include codes like Deputy of Health in the UMSs, Lack of a single instruction, The haste in concluding partnership contracts, Weakness in the regulated partnership contracts, Weakness in the expert capacity required for drafting partnership contracts, and Viewpoints inconsistency on the setting of partnership contracts. Provider payment includes codes like Deputy of Health in the UMSs, Existence of instructions, Delays in fulfilling government obligations, Low-profit margins, and Sustainability of financial resources.

Navigate back to Table 1..
